# Image-Based Airborne Sensors: A Combined Approach for Spectral Signatures Classification through Deterministic Simulated Annealing

**DOI:** 10.3390/s90907132

**Published:** 2009-09-08

**Authors:** María Guijarro, Gonzalo Pajares, P. Javier Herrera

**Affiliations:** 1 Ingeniería Técnica en Informática de Sistemas, Centro Superior de Estudios Felipe II, 28300 Aranjuez, Madrid, Spain; 2 Departamento de Ingeniería del Software e Inteligencia Artificial, Facultad Informática, Universidad Complutense, 28040 Madrid, Spain; E-Mail: pjherrera@pdi.ucm.es

**Keywords:** deterministic simulated annealing, image-based airborne sensors, classifier combination, fuzzy classifier, Bayesian classifier, unsupervised, spectral signatures classification

## Abstract

The increasing technology of high-resolution image airborne sensors, including those on board Unmanned Aerial Vehicles, demands automatic solutions for processing, either on-line or off-line, the huge amountds of image data sensed during the flights. The classification of natural spectral signatures in images is one potential application. The actual tendency in classification is oriented towards the combination of simple classifiers. In this paper we propose a combined strategy based on the Deterministic Simulated Annealing (*DSA*) framework. The simple classifiers used are the well tested supervised parametric Bayesian estimator and the Fuzzy Clustering. The *DSA* is an optimization approach, which minimizes an energy function. The main contribution of *DSA* is its ability to avoid local minima during the optimization process thanks to the annealing scheme. It outperforms simple classifiers used for the combination and some combined strategies, including a scheme based on the fuzzy cognitive maps and an optimization approach based on the Hopfield neural network paradigm.

## 1. Introduction

Nowadays the increasing technology of airborne sensors with their capabilities for capturing images, including those on board the new generations of Unmanned Aerial Vehicles, demands solutions for different image-based applications. Natural spectral signature classification is one of such applications because of the high image spatial resolution. The areas where the identification of spectral signatures are suitable include agricultural crop ordination, forest areas determination, urban identification and damage evaluation in catastrophes or dynamic path planning during rescue missions or intervention services also in catastrophes (fires, floods, etc.), among others. This justifies the choice of the images with different spectral signatures as the data where the proposed approach is to be applied, providing an application for this kind of sensors.

All classification problems need the selection of features to be classified and their associated attributes or properties, where a feature and its attributes describe a pattern. The behaviour of different features has been studied in texture classifications [1–3]. There are two categories depending on the nature of the features used: pixel-based [4–6] and region-based [2,7–10]. A pixel-based classification tries to classify each pixel as belonging to one of the clusters. The region-based identifies patterns of textures within the image and describes each pattern by applying filtering (laws masks, Gabor filters, wavelets, etc.), it is assumed that each texture displays different levels of energy allowing its identification at different scales. The aerial images used in our experiments do not display texture patterns. This implies that textured regions cannot be identified. In this paper we focus on the pixel-based category. Taking into account that we are classifying multi-spectral textured images, we use as attributes the three visible spectral Red-Green-Blue components, i.e., the RGB colour mapping. The RGB map performs better than other colour representations [11]; we have verified this assertion in our experiments, justifying its choice.

An important issue reported in the literature is that the combination of classifiers performs better than simple classifiers [1,12–16]. Particularly, the studies in [17] and [18] report the advantages of using combined classifiers against simple ones. This is because each classifier produces errors on a different region of the input pattern space [19].

Nevertheless, the main problem is: what strategy to choose for combining individual classifiers? This is still an open issue. Indeed in [13] it is stated that the same method can work appropriately in one application and produce poor results in another. Hence, our goal is to find a combined strategy that works conveniently for classifying spectral signatures in images. In [15] and [20] a revision of different approaches is reported including the way in which the classifiers are combined. Some important conclusions are: 1) if only labels are available, a majority vote should be suitable; 2) if continuous outputs like posterior probabilities are supplied, an average or some other linear combinations are suggested; 3) if the classifier outputs are interpreted as fuzzy membership values, fuzzy approaches, such as aggregation operators, could be used; 4) also it is possible to train the output classifier separately using the outputs of the input classifiers as new patterns, where a hierarchical approach can be used [1].

We propose a new approach which combines two individual classifiers: the probabilistic *parametric Bayesian* (*BP*) approach [21] and the *fuzzy clustering* (*FC*) [21,22]. The following two phases are involved during any classification process: training and decision. Really, the combination of the outputs provided by the two individual classifiers is carried out during the decision phase, as we will explain later. Given a set of training data, scattered through the tri-dimensional RGB data space and assuming known the number of clusters and the distribution of the samples into the clusters, both *BP* and *FC* individual classifiers estimate their associated parameters. Based on these estimated parameters, during the decision phase, each individual classifier provides for each pixel to be classified, a support of belonging to a cluster, *BP* provides probabilities and *FC* membership degrees, i.e., continuous outputs.

Because the number of classes is known, we build a network of nodes *net_j_* for each class *w_j_*, where each node *i* in the *net_j_* is identified as a pixel location *i* ≡ (*x*, *y*) in the image which is to be classified. Each node *i* is initialized in the *net_j_* with the output probability, provided by *BP*, that the node belongs to the class *w_j_*. This is the initial state value for the node *i* in the *net_j_*. Each state is later iteratively updated through the Deterministic Simulated Annealing (*DSA*) optimization strategy taking into account the previous states and two types of external influences exerted by other nodes on its neighbourhood. The external influences are mapped as consistencies under two terms: regularization and contextual. These terms are clique potentials of an underlying Markov Random Field model [23] and they both involve a kind of human perception. Indeed, the tri-dimensional scenes are captured by the imaging sensor and mapped in the bi-dimensional space, although the third dimension is lost under this mapping, the spatial grouping of the regions is preserved, and they are visually perceived grouped together like in the real scene.

The above allows the application of the Gestalt principles of psychology [24,25], specifically: similarity, proximity and connectedness. The similarity principle states that similar pixels tend to be grouped together. The proximity principle states that pixels near to one another tend to be grouped together. The connectedness states that the pixels belonging to the same region are spatially connected. The proximity and connectedness principles justify the choice of the neighbourhood for defining the regularization and contextual terms and the similarity establishes the analogies in the supports received by the pixels in the neighbourhood coming from the individual classifiers. From the point of view of the combination of classifiers the most relevant term is the regularization one. This is because it compares the supports provided by the individual classifier *FC* as membership degrees and the states of the nodes in the networks, which, as aforementioned, initially are the probabilities supplied by the individual classifier *BP* as supports. Therefore, this is the term where the combination of classifiers is really carried out making an important contribution of this paper.

The choice of *BP* and *FC* as the simple classifiers for the combination is based on their well tested performance in the literature and also in the possibility of combining continuous outputs during the decision phase under a mechanism different from the classical one used in [15]. Nevertheless, different classifiers providing continuous outputs or some others where this can be obtained could be used. As mentioned before, we have focused the combination on the decision phase; this implies that other strategies that apply the combination based on the training one are out of the scope of this paper. One of them is proposed in [26], which has been used in various classification problems. In this model, a selector makes use of a separate classifier, which determines the participation of the experts in the final decision for an input pattern. This architecture has been proposed in the neural network context. The experts are neural networks, which are trained so that each network is responsible for a part of the feature space. The selector uses the output of another neural network called the gating network [15]. The input of the gating network is the pattern to be classified and the output is a set of outputs determining the competences for each expert. These competences are used together during the decision with the classifier outputs provided by the experts. Under the above considerations we justify the choice of *BP* and *FC* as the base classifiers for the proposed combined strategy.

We have designed similar combined strategies. The first one is based on the fuzzy cognitive maps (*FCM*) framework [27] and the second in the analog Hopfield neural network (*HNN*) paradigm [28], where in the latter an energy minimization approach is also carried out. The best performance achieved, considering both strategies, is about an 85% success. After additional experiments with the *HNN*, we have verified that this is because the energy falls some times in local minima that are not global optima. This behaviour of *HNN* is reported in [29]. The *DSA* is also an energy optimization approach with the advantage that it can avoid local minima. Indeed, according to [23] and reproduced in [29], when the temperature involved in the simulated annealing process satisfies some constraints (explained in the section 2.2) the system converges to the minimum global energy which is controlled by the annealing scheduling instead of the nonlinear first-order differential equation used in *HNN*. This is the main difference of the proposed *DSA* technique with respect to the *HNN* approach. The *FCM* does not work with energy minimization, but because it does not improve the results of *HNN*, we think that it is unable to solve this problem. Hence, we exploit the capability of the *DSA* for avoiding local minima, making the main contribution of this paper. The *DSA* outperforms the *FCM* and *HNN* combined strategies, also the classical ones and the simple classifiers.

The paper is organized as follows. In Section 2 we give details about the proposed combined classifier, describing the training and decision phases, specially the last one where the *DSA* mechanism is involved. In Section 3 we give details about the performance of the proposed strategy applied to natural images displaying different spectral signatures. Finally, the conclusions are presented in Section 4.

## 2. Design of the Classifier

The system works in two phases: training and decision. As mentioned before, we have available a set of scattering patterns for training, partitioned into a known number of classes, *c*. With such purpose, the training patterns are supplied to the *BP* and *FC* classifiers for computing their parameters. These parameters are later recovered during the decision phase for making decisions about the new incoming samples, which are to be classified.

### 2.1. Training Phase

During the training phase, we start with the observation of a set *X* of *n* training samples, i.e., *X* = {***x***_1_,***x***_2_,…,***x****_n_*} ∈ ℜ*^d^*, where *d* is the data dimensionality, which is set to 3 because the samples represent the R,G and B spectral components of each pixel. Each sample is to be assigned to a given class *w_j_*, where the number of possible classes is *c*, i.e., *j* = 1, 2,…,*c*.

#### a) Fuzzy Clustering (FC)

This process receives the input training patterns and computes for each ***x****_i_* ∈ *X* at the iteration *t* its membership grade $\mu_{i}^{j}$ and updates the class centres, ***v****_j_* ∈ ℜ*^d^* as follows [20,22]:

(1) $$\mu_{i}^{j}\;(t+1)=\frac{1}{\sum_{r=1}^{c}\;{\left(d_{\mathrm{ij}}\;(t)/d_{\mathrm{ir}}\;(t)\right)}^{2/(m-1)}};\;\;v_{j}\;(t+1)=\frac{\sum_{i=1}^{n}\;\mu_{i}^{j}\;{\left(t\right)}^{m}\;x_{i}}{\sum_{i=1}^{n}\;\mu_{i}^{j}\;{\left(t\right)}^{m}}$$

$d_{\mathrm{ij}}^{2}=d^{2}\left(x_{i},\;v_{j}\right)$ is the squared Euclidean distance between ***x****_i_* and ***v****_j_* and equivalently $d_{\mathrm{ir}}^{2}$ between ***x****_i_* and ***v****_r_*. The number *m* is called the exponent weight [22,30]. The stopping criterion of the iteration process is achieved when $\left\|\mu_{i}^{j}\;(t+1)-\mu_{i}^{j}\;(t)\right\|<\varepsilon \;\;\forall \;\mathrm{ij}$ or a number *t_max_* of iterations is reached, set to 50 in our experiments; *ε* has been fixed to 0.01 after experimentation. Once the fuzzy clustering process is carried out, each class *w_j_* has associated its centre ***v****_j_*.

#### b) Bayesian Parametric (BP) estimation

Assuming known the distribution (Gaussian) for each class *w_j_*, the probability density function is expressed as follows:

(2) $$p\left(x|w_{j}\right)=\frac{1}{{\left(2\pi \right)}^{d/2}{\left|C_{j}\right|}^{1/2}}\;\text{exp}\;\left[-\frac{1}{2}\;{\left(x-m_{j}\right)}^{t}\;\;C_{j}^{-1}\;\left(x-m_{j}\right)\right]$$

where the parameters to be estimated are the mean ***m****_j_* and the covariance *C_j_*, both for each class *w_j_* with *n_j_* samples. They are estimated through maximum likelihood as given by equation (3):

(3) $$m_{j}=\frac{1}{n_{j}}\;\sum_{k=1}^{n_{j}}\;x_{k}\;\;\;\;C_{j}=\frac{1}{n_{j}-1}\;\sum_{k=1}^{n_{j}}\;\left(x_{k}-m_{j}\right)\;{\left(x_{k}-m_{j}\right)}^{T}$$

where *T* denotes transpose. The parameters ***v****_j_*, ***m****_j_* and *C_j_* are stored to be recovered during the next decision phase.

### 2.2. Decision Phase

Given a new sample ***x****_i_*, the problem is to decide which the cluster it belongs is. We make the decision based on the final state values after the *DSA* optimization process. As mentioned before, the *DSA* is an energy optimization based approach with the advantage that it can avoid local minima. Indeed, in accordance with [23] and reproduced in [29], when the temperature involved in the simulated annealing process satisfies some constraints, explained below, the system converges to the minimum global energy which is controlled by the annealing. The minimization is iteratively achieved by modifying the state of each node through the external influences exerted by other nodes and its own state on the previous iteration.

As mentioned during the introduction, for each cluster *w_j_*, we build a network of nodes, *net_j_*. Each node *i* in the *net_j_* is associated to the pixel location *i* ≡ (*x*, *y*)in the image, which is to be classified; the node *i* in the *net_j_* is initialized with the probability $p_{i}^{j}\equiv p\;\left(x_{i}|w_{j}\right)$ provided by *BP* according to the equation (2), but mapped linearly to the range [−1,+1] instead of [0,+1]. The probabilities are the initial network states associated to the nodes. As it is known, the simple *BP* method classifies each pixel *i* as belonging to the cluster *w_j_* according to the maximum network state value associated to the pixel *i* in the *j* networks, i.e., *i* ∈ *w_j_* if $p_{i}^{j}>p_{i}^{h}$, ∀ *j* ≠ *h*. Through the *DSA* these network states are reinforced or punished iteratively based on the influences exerted by their neighbours. The goal is to make better decisions based on more stable state values.

Suppose a network with *N* nodes. The simulated annealing optimization problem is: modify the analogue values $p_{i}^{j}$ so as to minimize the energy [21,29]:

(4) $$E=-\frac{1}{2}\;\sum_{j=1}^{c}\;\sum_{i=1}^{N}\;\sum_{k=1}^{N}\;s_{\mathrm{ik}}^{j}\;p_{i}^{j}\;p_{k}^{j}$$

where $s_{\mathrm{ik}}^{j}$ is the symmetric weight interconnecting two nodes *i* and *k* in the *net_j_* and can be positive or negative ranging in [−1,+1]; $p_{k}^{j}$ is the state of the neighbouring node *k* in the *net_j_*. Each $s_{\mathrm{ik}}^{j}$ determines the influence that the node *k* exerts on *i* trying to modify the state $p_{i}^{j}$. According to [21] the self-feedback weights must be null (i.e., $s_{\mathrm{ii}}^{j}=0$). The *DSA* approach tries to achieve the most network stable configuration based on the energy minimization. From equation (4) one can see that this expression requires the computation of $s_{\mathrm{ik}}^{j}$ and the states of the nodes $p_{i}^{j}$ and $p_{k}^{j}$ ; $s_{\mathrm{ik}}^{j}$ will be defined later in the equation (7); both $p_{i}^{j}$ and $p_{k}^{j}$ are obtained after the corresponding updating process.

The term $s_{\mathrm{ik}}^{j}$ is a combination of two coefficients representing the mutual influence exerted by the *k* neighbours over *i*, namely: *a*) a *regularization* coefficient which computes the consistency between the states of the nodes and the membership degrees provided by *FC* in a given neighbourhood for each *net_j_*; *b*) a *contextual* coefficient which computes the consistency between the class labels obtained after a previous classification phase. Both consistencies are based on the similarity Gestalt’s principle [24,25], as explained in the introduction. The neighbourhood is defined as the *m*-connected spatial region, $N_{i}^{m}$, where *m* is set to 8 in this paper and allows the implementation of the proximity and connectedness Gestalt’s principles [24,25], also explained in the introduction The regularization coefficient is computed at the iteration *t* according to the equation (5):

(5) $$r_{\mathrm{ik}}^{j}\;(t)=\{\begin{array}{ll} 1-\left|p_{i}^{j}\;(t)-\mu_{k}^{j}\right| & k\in N_{i}^{m},\;\;i\neq k\\ 0 & k\notin N_{i}^{m}\;\;\mathrm{or}\;\;\;i=k \end{array}$$

where $\mu_{k}^{j}$ is the membership degree, supplied by *FC*, that a node (pixel) *k* with attributes ***x****_k_* belongs to the class *w_j_*, computed through the equation (1). These values are also mapped linearly to range in [−1,+1] instead of [0,+1]. From (5) we can see that $r_{\mathrm{ik}}^{j}\;(t)$ ranges in [−1,+1] where the lower/higher limit means minimum/maximum influence respectively.

The contextual coefficient at the iteration *t* is computed taking into account the class labels *l_i_* and *l_j_* as follows, where values of −1 and +1 mean negative and positive influence respectively:

(6) $$c_{\mathrm{ik}}\;(t)=\{\begin{array}{lll} +1 & l_{i}(t)=l_{k}(t) & k\in N_{i}^{m},\;\;\;i\neq k\\ -1 & l_{i}(t)\neq l_{k}(t) & k\in N_{i}^{m},\;\;\;\;i\neq k\\ 0 & & k\notin N_{i}^{m},\;\;\;i=k \end{array}$$

Labels *l_i_* and *l_k_* are obtained as follows: given the node *i*, at each iteration *t*, we know its state at each *net_j_* as given by the next equation (8), initially through the supports provided by *BP*; we determine that the node *i* belongs to the cluster *w_j_* if $p_{i}^{j}>p_{i}^{h}$, ∀ *j*≠*h*, so we set *l_i_* to the *j* value which identifies the cluster, *j* = 1,..., *c*. The label *l_k_* is set similarly. Thus, this coefficient is independent of the *net_j_*, because it is the same for all networks. Both coefficients are combined as the averaged sum, taking into account the signs:

(7) $$W_{\mathrm{ik}}^{j}\;(t)=\gamma r_{\mathrm{ik}}^{j}\;(t)+(1-\gamma )\;c_{\mathrm{ik}}\;(t);\;\;\;\;s_{ik}^{j}={\left[\mathrm{sgn}\left(W_{\mathrm{ik}}^{j}\right)\right]}^{v}\;W_{\mathrm{ik}}^{j};\;\;\;\;\;\mathrm{sgn}\left(W_{\mathrm{ik}}^{j}\right)=\{\begin{array}{ll} -1 & W_{\mathrm{ik}}^{j}\leq 0\\ +1 & W_{\mathrm{ik}}^{j}>0 \end{array}$$

*γ* ∈ [0,1] represents the trade-off between both coefficients. After a set of experiments we have chosen *γ* = 0.80 because *c_ik_*(*t*) considers the state values which are directly involved in the energy computation through the equation (4). This avoids the over contribution of the state values in the energy value; *sgn* is the *signum function* and *v* is the number of negative values in the set $C\equiv \left\{W_{\mathrm{ik}}^{j}\;(t),\;r_{\mathrm{ik}}^{j}\;(t),\;c_{\mathrm{ik}}\;(t)\right\}$, i.e., given *S* ≡ {*q* ∈ *C*/*q* < 0} ⊆ *C*, *v* = card (*S*). Note that *c_ik_*(*t*) after a previous decision phase.

The simulated annealing process was originally developed in [31,32] under a stochastic approach. In this paper we have implemented the deterministic one described in [21,33] because, as reported here, the stochastic is slow due to its discrete nature as compared to the analogue nature of the deterministic. Following the notation in [21], let $u_{i}^{j}\;(t)=\sum_{k}\;s_{\mathrm{ik}}^{j}\;(t)p_{k}^{j}\;(t)$ be the force exerted on node *i* by the other nodes $k\in N_{i}^{m}$ at the iteration *t*; then the new state $p_{i}^{j}\;(t+1)$ is obtained by adding the fraction *f*(·,·) to the previous one as follows:

(8) $$p_{i}^{j}\;(t+1)=\frac{1}{2}\;\left[f\;\left(u_{i}^{j}\;(t),T(t)\right)+p_{i}^{j}\;(t)\right]=\frac{1}{2}\left[\tanh\left(u_{i}^{j}\;(t)/T\;(t)\right)+p_{i}^{j}\;(t)\right]$$

Where, as always, *t* represents the iteration index. The fraction *f*(·,·) depends upon $u_{i}^{j}\;(t)$ and the temperature *T* at the iteration *t*.

The equation (8) differs from the updating process in [21] because we have added the term $p_{i}^{j}\;(t)$ to the fraction *f*(·,·). This modification represents the contribution of the self-support from node *i* to its updating process. This implies that the updated value for each node *i* is obtained by taking into account its own previous state value and also the previous state values and membership degrees of its neighbours. The introduction of the self support tries to minimize the impact of an excessive neighbouring influence. Hence, the updating process tries to achieve a trade-off between its own influence and the influence exerted by the nodes *j* by averaging both values.

One can see from equation (7) that if a node *i* is surrounded by nodes with similar state values and labels, $s_{\mathrm{ik}}^{j}\;(t)$ should be high. This implies that the $p_{i}^{j}\;(t)$ value should be reinforced through equation (8) and the energy given by equation (4) is minimum and *vice versa*. Moreover, at high *T,* the value of *f*(·,·) is lower for a given value of the forces $u_{i}^{j}\;(t)$. Details about the behaviour of *T* are given in [21]. We have verified that the fraction $u_{i}^{j}\;(t)/T\;(t)$ must be small as compared to $p_{i}^{j}\;(t)$ in order to avoid that the updating is controlled only by $u_{i}^{j}\;(t)$. Under the above considerations and based on [23,30,33], the following annealing schedule suffices to obtain a global minimum: *T*(*t*) = *T*_0_/log(*t*+1), with *T*_0_ being a sufficiently high initial temperature. *T*_0_ is computed as follows [34]: 1) we select four images to be classified, computing the energy in (4) for each image after the initialization of the networks; 2) we choose an initial temperature that permits about 80% of all transitions to be accepted (i.e., transitions that decrease the energy function), and the temperature value is changed until this percentage is achieved; 3) we compute the *M* transitions Δ*E_k_* and we look for a value for *T* for which $\frac{1}{M}\;\sum_{k=1}^{M}\;\text{exp}\left(-\Delta E_{k}/T\right)=0.8$, after rejecting the higher order terms of the Taylor expansion of the exponential, *T* = 8〈Δ*E_k_*〉, where 〈·〉 is the mean value. In our experiments, we have obtained 〈Δ*E_k_*〉 = 1.22, giving *T*_0_ = 9.76 (with a similar order of magnitude as that reported in [33]). We have also verified that a value of *t_max_* = 200 suffices, although the expected condition *T*(*t*) = 0, *t* → +∞ in the original algorithm is not fully fulfilled. The assertion that it suffices is based on the fact that this limit was never reached in our experiments as shown later in the section 3, hence this value does not affect the results. The *DSA* process is synthesized as follows [21]:

1. *Initialization*: load each node with $p_{i}^{j}\;(t=0)$ according to the equation (2); set *ε* = 0.01 (constant to accelerate the convergence, section 3.1); *t_max_* = 100. Define *nc* as the number of nodes that change their state values at each iteration.
2. *DSA process*:
  t = 0
  *while t < t_max_* *or nc* ≠ 0
  *t* = *t* + 1; *nc* = 0;
  *for* each node *i*
    update $p_{i}^{j}\;(t)$ according to the equation (8) from equations (5) to (7)
    *if* $\left|p_{i}^{j}\;(t)-p_{i}^{j}\;(t-1)\right|>\varepsilon$
    *then*
      *nc* = *nc* + 1; *else nc* = *nc*
    end if; end for; end while
3. *Outputs*: the states $p_{i}^{j}\;(t)$ for all nodes updated.

The decision about the classification of a node *i* with attributes ***x****_i_* as belonging to the class *w_j_* is made as follows: *i* ∈ *w_j_* if $p_{i}^{j}>p_{i}^{h}$, ∀ *w_j_* ≠ *w_h_*.

## 3. Comparative Analysis and Performance Evaluation

To assess the validity and performance of the proposed approach we describe the tests carried out according to both processes: training and classification. First, we give details about the setting of some free parameters involved in the proposed method.

### 3.1. Setting Free Parameters

We have used several data sets for setting the free parameters; these are: 1) nine data sets from the Machine Learning Repository [35]: (bupa, cloud, glass, imageSegm, iris, magi4, thyroid, pimaIndians and wine); 2) three synthetic data sets manually generated with different numbers of classes and 3) four data sets coming from outdoor natural images, also with different numbers of classes. The use of these data, some of them different from the images with different spectral signatures, is justified under the idea that the values of the parameters to be set must have so much general validity as it is possible.

#### a) Parameters involved in the FC training phase

They are the exponential weight *m* in equation (1) and the convergence parameters *ε* and *t_max_* used for its convergence. The number of classes and the distribution of the patterns on the clusters are assumed to be known. We apply the following cross-validation procedure [21]. We randomly split each data set into two parts. The first (90% of the patterns) is used as the training set. The other set (validation set) is used to estimate the global classification error based on the single *FC* classifier. We set *m* = 2.0 (which is a usual value) and vary *ε* from 0.01 to 0.1 in steps of 0.015 and estimate the cluster centres and membership degrees for each training set. Then, we compute the error rate for each validation set. The maximum error was obtained with *ε* = 0.1 for 10 iterations and the minimum with *ε* = 0.01 and 47 iterations. Fixed those values, we vary *m* from 1.1 to 4.0 in steps of 0.1 and estimate once again the cluster centres and the membership degrees with the training set. Once again the validation sets are used for computing the error rates, the minimum error value is obtained for *m* = 2.0. The settings are finally fixed to *m* = 2.0, *ε* = 0.01 and *t_max_* = 50 (expanding the limit of 47).

#### b) DSA convergence

The *ε* used for accelerating the convergence in the *DSA* optimization approach is set to 0.01 by using the validation set for the four data sets coming from the outdoor natural images mentioned above. Verifying, that *t_max_* = 20 suffices.

### 3.2. Training Phase

We have available a set of 36 digital aerial images acquired during May in 2006 from the Abadin region located at Lugo (Spain). They are images in the visible range of the spectra, i.e., red-green-blue, 512 × 512 pixels in size. The images were taken during different days from an area with several natural spectral signatures. We select randomly 12 images from the set of 36 available. Each image is down sampled by two, eliminating a row and column of every two; so, the number of training samples provided by each image is the number of pixels. The total number of training samples is *n* = 12 × 256 × 256 = 786,432.

We have considered that the images have four clusters, i.e., *c* = 4. Table 1 displays the number of patterns used for training and the cluster centres estimated by the individual classifiers, which are ***v****_i_* for *FC* and ***m****_i_* for *BP*, equations (1) and (3) respectively.

### 3.3. Decision Phase and Comparative Analysis

The remaining 24 images from the set of 36 are used as images for testing. Four sets, S0, S1 S2 and S3 of six images each, are processed during the test according to the strategy described below. The images assigned to each set are randomly selected from the 24 images available.

#### a) Design of a test strategy

In order to assess the validity and performance of the proposed approach we have designed a test strategy with two purposes: 1) to verify the performance of our approach as compared against some existing strategies (simple and combined); 2) to study the behaviour of the method as the training (i.e., the learning) increases.

Our proposed combined *DSA* (DS) method is compared against the base classifiers used for the combination (*BP* and *FC*). It is also compared against the following classical combiners that apply the decision as described immediately after [15,20]. Consider the pixel *i* to be classified. *BP* and *FC* provide the probability $p_{i}^{j}$ and membership degree $\mu_{i}^{j}$ respectively, that the pixel *i* belongs to the class *w_j_*. After applying a rule, a new support $s_{i}^{j}$ is obtained for that pixel of belonging to *w_j_* as follows: *a*) Mean rule (ME) $s_{i}^{j}=\left(\mu_{i}^{j}+p_{i}^{j}\right)/2$; *b*) Maximum rule (MA) $s_{i}^{j}=\max\left\{\mu_{i}^{j},\;p_{i}^{j}\right\}$; *c*) Minimum rule (MI) $s_{i}^{j}=\min\left\{\mu_{i}^{j},\;p_{i}^{j}\right\}$ and *d*) Product rule (PD) $s_{i}^{j}=\mu_{i}^{j}\;p_{i}^{j}$. These rules have been studied in terms of reliability [36]. Yager [37] proposed a multi-criteria decision making approach based on fuzzy sets aggregation. It follows the general rule and the scheme of the combiners described in [21]. So, DS is also compared against the fuzzy aggregation (FA) where the final support that the pixel *i* belongs to the class *w_j_* is given by the following aggregation rule:

(9) $$s_{i}^{j}=1-\min\left\{1,{\left({\left(1-\mu_{i}^{j}\right)}^{a}+{\left(1-p_{i}^{j}\right)}^{a}\right)}^{1/a}\right\}\;\;\;\;\;\;\;\;\;a\geq 1$$

The parameter *a* has been fixed to 4 by applying a cross-validation procedure as the described in section 3.1*a*). Given the supports, according to each rule, the decision about the pixel *i* is made as follows: *i* ∈ *w_j_* *if* $s_{i}^{j}>s_{i}^{k}\;\forall w_{k}|\;w_{k}\neq w_{j}$.

Finally, and what it is more important, *DS* is compared against the optimization strategy based on the Fuzzy cognitive Maps (*FM*) [27] and the Hopfield neural Network (*HN*) [28] paradigms. Both are based on the same network topology like the used in this paper and compute the regularization and contextual coefficients similarly to the proposed in this paper through the equations (5) and (6), but using the membership degrees provided by *FC* for the networks initializations. Nevertheless, for comparison purposes, we have changed the roles in the experiments carried out here, so that the nodes in both *FM* and *HN* are initially loaded with the probabilities as in the proposed *DS* approach.

In order to verify the behaviour of each method as the learning degree increases, we have carried out the experiments according to the following three STEPs described below

STEP 1: given the images in S0 and S1, classify each pixel as belonging to a class, according to the number of classes established during the training phase. Compute the percentage of successes according to the ground truth defined for each class at each image. The classified pattern samples from S1 are added to the previous training samples and a new training process is carried out (Section 2.1) with the same number of clusters. The parameters associated to each classifier are updated. The set S0 is used as a pattern set in order to verify the performance of the training process as the learning increases. Note that it is not considered for training.

STEPs 2 and 3: perform the same process but using the sets S2 and S3 respectively instead of S1; S0 is also processed as before.

As one can see the number of training samples added at each STEP is 6 × 512 × 512 because this is the number of pixels classified during the STEPs 1 to 3 belonging to the sets S1, S2 and S3.

To verify the performance for each method we have built a ground truth for each image processed under the supervision of expert human criteria. Based on the assumption that the automatic training process determines four clusters, we classify each image pixel with the simple classifiers obtaining a labelled image with four expected clusters, and then we select the image with the best results, always according to the expert.

The labels for each cluster, from the selected labelled image, are manually touched up until a satisfactory classification is obtained under the human supervision. This implies that each pixel has assigned a unique label in the ground truth, which serves as the reference one for comparing the performances.

**Figure 1.**
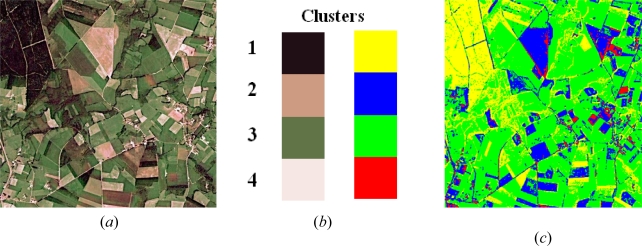
(*a*) original image belonging to the set S0; (*b*) correspondence between classes and labels; (*c*) labelled image with the four classes according to the labels in (*b*).

Figure 1(*a*) displays an original image belonging to the set S0; Figure 1(*b*) displays the correspondence between clusters and labels, in the left column the colour according to the values of the corresponding cluster centre and in the right column the artificial colour labels, both in the tri-dimensional RGB colour space; (*c*) labelled image for the four clusters obtained by our proposed *DS* approach.

The correspondence between labels and the different spectral signatures is: 1.-yellow, forest vegetation displaying obscure tones; 2.-blue, ochre tones without the spectral saturation of the sensor; 3.-green, agricultural crop vegetation; 4.-red, ochre tones with a clear tendency towards the spectral saturation of the sensor. In clusters 3 and 4 are included buildings, man made structures and also bare soils.

**Figure 2.**
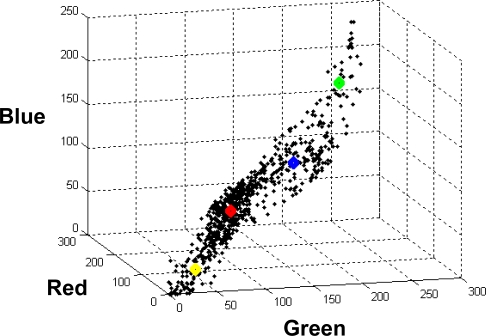
Distribution of a subset of 4,096 patterns into the four estimated classes around the cluster centres of the classes in the colour space RGB. The centres are displayed in the same colour as the labels in Figure 1(*b*).

Figure 2 displays the distribution of a representative subset of 4,096 patterns from the image of the Figure 1(*a*), obtained by down sampling the image by eight, into the clusters in the tri-dimensional RGB colour space, where the centres of the classes, obtained through the *BP* classifier during the training phase, are also displayed; they are the four ***m****_j_* cluster centres, displayed in the same colour as the labels in the Figure 1(*b*). As one can see, there is no a clear partition into the four clusters because the samples appear scattered in the whole space following the diagonal. Hence, the classification of the borders patterns becomes a difficult task because they can belong to more than one cluster depending on their proximity to the centres.

#### b) Results

Table 2 shows the percentage of error during the decision for the different classifiers. For each STEP from 1 to 3, we show the results obtained for both sets of tested images S0 and either S1 or S2 or S3.

These percentages are computed as follows. Let $I_{N}^{r}$ an image *r* (*r* = 1,…,6) belonging to the set *SN* (*N* = 0,1,2,3); *i* is the node at the pixel location (*x*,*y*) in $I_{N}^{r}$. An error counter $E_{N}^{r}$ is initially set to zero for each image *r* in the set *SN* at each STEP and for each classifier. Based on the corresponding decision process, each classifier determines the class to which the node *i* belongs, *i* ∈ *w_j_*. If the same pixel location on the corresponding ground truth image is black then the pixel is incorrectly classified and $E_{N}^{r}=E_{N}^{r}+1$. The error rate of the image $I_{N}^{r}$ is: $e_{N}^{r}=E_{N}^{r}/Z$, where *Z* is the image size, i.e., 512 × 512. The average error rate for the set *SN* at each STEP is given by:

(10) $${\bar{e}}_{N}=\frac{1}{6}\;\sum_{r=1}^{6}\;e_{N}^{r}$$

and the standard deviation by:

(11) $${\bar{\sigma }}_{N}=\sqrt{\frac{1}{5}\;\sum_{r=1}^{6}\;{\left(e_{N}^{r}-{\bar{e}}_{N}\right)}^{2}}$$

In the Table 2 they are displayed as percentages, i.e., *ẽ_N_* = 100 *ē_N_* and *σ̃_N_* = 100 *σ̄_N_*. The numbers in square brackets indicate the rounded and averaged number of iterations required by DS, HN and FM for each set (S0, S1, S2 and S3) at each STEP (1, 2 and 3).

**Figure 3.**
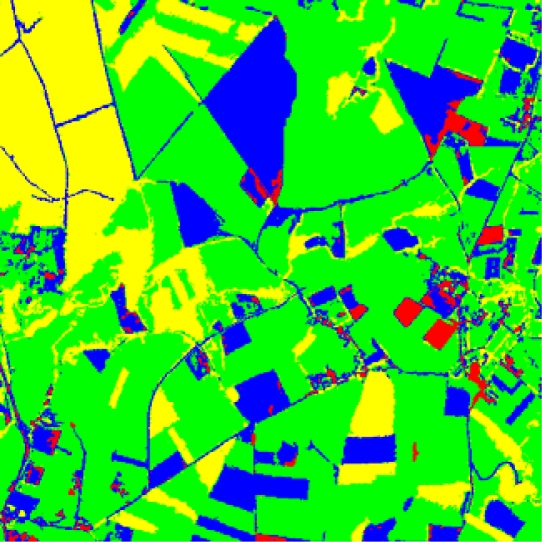
Ground truth image where the labels for the four clusters displayed in Figure 1 have been manually rectified.

Figure 3 displays the ground truth image for the one in Figure 1(*a*) which has been manually rectified from the results obtained through the *BP* classifier. As in the image of Figure 1(*c*), each colour identifies the corresponding label for the four clusters represented in Figure 1.

#### c) Discussion

Based on the error rates displayed in Table 2, we can see that in general, the proposed DS approach outperforms the other methods and achieves the less error rates for STEP 3 in both sets S0 and S1. All strategies achieve the best performance in the STEP 3. Of particular interest is the improvement achieved for the set S0 in STEP 3 with respect the results obtained in STEPs 1 and 2 for that set. Based on the above observations, we can conclude that the learning improves the results, i.e., better decisions can be made as the learning increases. A detailed analysis for groups of classifiers is the following:

1. *Simple classifiers*: the best performance is achieved by *BP* as compared to *FC*. This suggests that the network initialization, through the probabilities supplied by *BP*, is acceptable.
2. *Combined rules*: the mean and product rules achieve both similar averaged errors. The performance of the mean is slightly better than the product. This is because, as reported in [38], combining classifiers which are trained in independent feature spaces result in improved performance for the product rule, while in completely dependent feature spaces the performance is the same. We think that this occurs in our RGB feature space because of the high correlation among the R, G and B spectral components [39,40]. High correlation means that if the intensity changes, all the three components will change accordingly.
3. *Fuzzy combination*: this approach outperforms the simple classifiers and the combination rules. Nevertheless, this improvement requires the convenient adjusting of the parameter *a*, with other values the results get worse.
4. *Optimization and relaxation approaches*: once again, the best performance is achieved by *DS*, which with a similar number of iterations that *HN* obtains better percentages of successes, the improvement is about 3.6 percentage points. *DS* also outperforms *FM*. This is because *DS* avoids satisfactorily some minima of energy, as expected.

**Figure 4.**
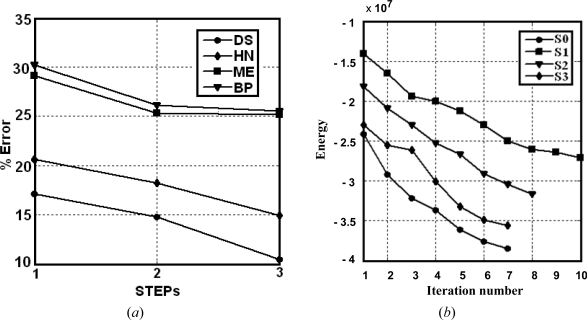
(*a*) percentage of error for DS, HN, ME and BP against the three STEPs; (*b*) energy behaviour for S0 to S3 against the number of iterations.

For clarity, in Figure 4(*a*) the performance of the proposed *DS* approach for the set S0 is displayed against *HN*, because both are optimization approaches based on energy minimization; *ME* which is the best method of the combination rules and *BP*, the best method of simple combiners. Figure 4(*b*) shows the energy behaviour for the four sets (S1, S2, S3 and S0 in STEP 3) against the averaged number of iterations required to reach the convergence. The energy decreases as the optimization process increases, as expected according to the equation (4). Similar slopes can be observed for the sets S0, S2 and S3. On the contrary, the slope for S1 is smoother; this explains the greater number of iterations required for this set during the convergence.

Overall, the results show that the combined approaches perform favourably for the data sets used. The MA and ME fusion methods also provide best results than the individual ones. This means that combined strategies are suitable for classification tasks. This agrees with the conclusion reported in [13] or [15] about the choice of combined classifiers. Moreover, as the learning increases through STEPs 1 to 3 the performance improves and the number of iterations for S0 decreases, because part of the learning has been achieved at this stage. This means that the learning phase is important and that the number of samples affects the performance.

The main drawback of the DS, as well as also for the HN and FM approaches, is its execution time, which is greater than the methods that do not apply relaxation processes. This is a general problem for all kind of relaxation or optimization approaches.

All tests have been implemented in MATLAB and executed on an Intel Core 2 Duo, 2.40 GHz PC with 2.87 GB RAM operating under Microsoft Windows XP service pack 3. On average, the execution time per iteration and per image is 10.1 seconds.

## 4. Conclusions

During the decision phase, we have proposed a combined strategy under the *DSA* framework performing favourably as compared against other existing combined strategies including those with similar design and based on optimization and also against the individual classifiers. The application of the similarity, proximity and connectedness Gestalt’s principles allows combining probabilities and membership degrees, supplied by the *BP* and *FC* classifiers respectively, by means of the regularization and contextual coefficients. The probabilities supplied by *BP* are used as initial states in a set of neural networks, which are specifically designed with such purpose. These states are iteratively updated under the *DSA* optimization process through the external influences exerted by the nodes in the neighbourhood thanks to the application of the Gestalt’s principles.

In future works the updating through the *DSA* of both probabilities and membership degrees could be considered. With the proposed combined approach, we have established the bases to be able for combine more than two classifiers. This can be made by re-defining the regularization coefficient.

Also, if we try to combine classifiers providing outputs in different ranges always it should be possible to map all outputs in the same range. This allows the combination of different kinds of classifiers including self-organizing maps or vector quantization with *BP* or *FC* by example.

## Figures and Tables

**Table 1.** Number of patterns used for training and class centres obtained for each class according to the simple classifiers FC and BP.

	**cluster *w*_1_**	**cluster *w*_2_**	**cluster *w*_3_**	**cluster *w*_4_**
**Number of patterns**	139,790	196,570	387,359	62,713
***BP* (*m_i_*)**	(37.5, 31.3, 21.5)	(167.0,142.6, 108.4)	(93.1, 106.0, 66.4)	(226.7, 191.9, 180.4)
***FC* (*v_i_*)**	(35.3, 28.8, 19.9)	(168.0,142.8,108.6)	(93.0, 106.4, 66.5)	(229.1, 194.0, 184.4)

**Table 2.** Average percentages of error and standard deviations at each STEP for the four sets of tested images S0, S1, S2 and S3.

*ẽ_N_*: average percentage of error *σ̃_N_*: standard deviation of error		**STEP 1**				**STEP 2**				**STEP 3**			
		**S0**		**S1**		**S0**		**S2**		**S0**		**S3**	
		*ẽ*_0_	*σ̃*_0_	*ẽ*_1_	*σ̃*_1_	*ẽ*_0_	*σ̃*_0_	*ẽ*_2_	*σ̃*_2_	*ẽ*_0_	*σ̃*_0_	*ẽ*_3_	*σ̃*_3_
**Combination by optimization (DS, HN) and relaxation (FM)**	**[iterations]** **DS** (Simulated)	**[8]** 17.1	1.1	**[10]** 17.8	1.2	**[8]** 14.8	1.0	**[8]** 13.8	0.8	**[7]** 10.5	0.7	**[7]** 13.5	0.7
	**[iterations]** **HN** (Hopfield)	**[9]** 20.6	1.6	**[10]** 21.5	1.5	**[9]** 18.2	1.2	**[8]** 17.2	1.0	**[7]** 14.9	0.8	**[8]** 17.2	0.8
	**[iterations]** **FM**(Fuzzy C.)	**[16]** 21.6	1.7	**[18]** 21.6	1.6	**[14]** 19.1	1.2	**[15]** 19.8	1.1	**[11]** 16.0	0.9	**[12]** 18.6	0.8
**Fuzzy Combination**	**FA** (Yager)	25.5	2.2	26.8	2.1	24.1	1.9	24.4	1.8	21.5	1.6	20.8	1.5
**Combination rules**	**MA** (Maximum)	31.2	2.9	30.7	2.7	28.4	2.8	27.5	2.6	26.9	2.1	26.8	1.9
	**MI** (Minimum)	37.1	3.1	36.9	2.9	32.2	3.3	35.2	2.8	30.9	2.4	28.5	2.3
	**ME** (Mean)	29.1	2.6	28.6	2.2	25.3	2.3	26.4	2.2	25.5	1.9	24.3	1.7
	**PR** (Product)	29.5	2.7	29.1	2.3	25.8	2.4	27.0	2.4	25.2	2.1	25.1	1.8
**Simple classifiers**	**BP** (Bayesian Parametric)	30.2	2.7	29.1	2.5	26.1	2.2	26.4	2.2	25.2	2.0	24.7	1.8
	**FC** (Fuzzy clustering)	32.1	2.8	30.2	2.6	27.1	2.3	27.4	2.3	26.0	2.1	25.9	2.0
